# Acute Coronary Occlusion in NSTEMI Patients: Prevalence, Clinical Characteristics and the Potential Role of Artificial Intelligence

**DOI:** 10.3390/medicina62050899

**Published:** 2026-05-07

**Authors:** Christina Stathakopoulou, Charalampos Varlamos, Haroun Butt, Iosif Xenogiannis, Vassiliki-Maria Dragona, Despoina-Rafailia Benetou, Stefanos Vlachos, Christos Pappas, Fotios Kolokathis, Thomas R. Keeble, Grigoris V. Karamasis

**Affiliations:** 1 Cardiology Department, Attikon University Hospital, Athens Medical School, National and Kapodistrian University of Athens, 12461 Athens, Greece; 2 Anglia Ruskin School of Medicine and MTRC, Anglia Ruskin University, Chelmsford CM1 1SQ, Essex, UK; 3 Essex Cardiothoracic Centre, Basildon SS16 5NL, Essex, UK

**Keywords:** acute coronary occlusion, non-ST-elevation myocardial infarction (NSTEMI), artificial intelligence

## Abstract

*Background and Objectives*: The electrocardiogram (ECG)–based STEMI/NSTEMI classification determines the urgency of invasive management in acute myocardial infarction. However, it often underestimates the presence of acute coronary occlusion (ACO) in patients presenting with non-ST-elevation myocardial infarction (NSTEMI). Artificial intelligence (AI)-assisted ECG interpretation has emerged as a potential tool to improve early recognition of ACO. This study aimed to determine the prevalence of ACO among NSTEMI patients, to compare clinical characteristics between patients with and without ACO and to explore the potential role of AI in earlier recognition of ACO. *Materials and Methods*: All consecutive NSTEMI patients undergoing coronary angiography between September 2022 and December 2024 were included. Contrary to other studies that included TIMI flow grades 0–1, 0–2, or 0–3, ACO in our study was defined strictly as a culprit lesion with TIMI flow grade 0 at index coronary angiography. Clinical characteristics were compared between ACO and non-ACO patients. Admission 12-lead ECGs from ACO patients were retrospectively analysed using a clinically validated AI-based ECG interpretation model and classified according to the urgency of invasive management. *Results*: Among 520 NSTEMI patients, 49 (9.4%) had angiographically confirmed ACO. Within the non-ACO group, 7.0% of patients had TIMI flow grade 1 on index coronary angiography (6.3% of the total population). Therefore, 15.7% of the study population had TIMI flow grade 0/1. ACO patients were younger (60.9 ± 12.8 vs. 66.3 ± 12.0 years, *p* = 0.0065). Clinical characteristics did not differ between the groups, except for dyslipidemia, which was more prevalent in non-ACO patients (38.8% vs. 53.9%, *p* = 0.043). Revascularisation rates were higher in the ACO group (93.9% vs. 82.2%, *p* = 0.037). Culprit vessel distribution differed markedly between the groups (*p* < 0.0001). In multivariable logistic regression analysis, age was independently associated with ACO (OR 0.96, 95% CI 0.93–0.99, *p* = 0.007). AI-assisted ECG analysis was performed in 42 ACO patients; 57.1% were classified as requiring immediate invasive management. *Conclusions*: A significant proportion of NSTEMI patients have ACO. AI-assisted ECG interpretation may support earlier identification of ACO, although its clinical impact requires further validation. Future studies are warranted to confirm these findings.

## 1. Introduction

The electrocardiogram (ECG)–based classification of acute myocardial infarction (AMI) into ST-elevation myocardial infarction (STEMI) and non-ST-elevation myocardial infarction (NSTEMI) remains central to clinical decision-making [1,2]. This binary framework has long guided the urgency of invasive management among patients presenting with AMI [2,3]. However, it often underestimates the presence of acutely occluded coronary arteries among patients classified as NSTEMI [4]. Emerging evidence indicates that a significant proportion of NSTEMI patients present with acute coronary occlusion (ACO), resulting in delayed invasive management, higher rates of major adverse cardiac events (MACE) and increased mortality [5,6,7,8,9,10,11]. Due to these limitations, an occlusion-oriented myocardial infarction paradigm has been proposed, focusing on the underlying pathophysiology rather than ST-segment elevation patterns [7,11,12].

The 2022 American College of Cardiology (ACC) Expert Consensus Decision Pathway on Chest Pain emphasised the need to broaden ECG interpretation criteria to improve early recognition of acute coronary occlusion and achieve timely reperfusion [13]. A plethora of ECG criteria have been proposed to improve diagnostic sensitivity for ACO compared with the current STEMI criteria [14,15,16,17,18,19]. However, in clinical practice, early identification of ACO remains challenging.

Given that time delays in primary angioplasty affect mortality [20], artificial intelligence (AI)-assisted ECG interpretation has recently been proposed to enhance diagnostic accuracy and sensitivity and to improve early recognition of ACO [12,21,22,23,24,25,26,27].

The present study aimed to determine the prevalence of ACO in patients presenting with NSTEMI and undergoing coronary angiography and to compare clinical characteristics between patients with and without ACO. Furthermore, it explored the potential contribution of artificial intelligence in earlier recognition of ACO.

## 2. Materials and Methods

**Study design and population:** The study was conducted using data from the prospective Catheterisation Laboratory database of Attikon University Hospital, Athens, Greece. All consecutive adult patients (≥18 years) presenting with NSTEMI and undergoing coronary angiography between September 2022 and December 2024 were included. STEMI/NSTEMI classification was based on the ESC guideline-recommended criteria and on the Fourth Universal Definition of Myocardial Infarction [1,2]. The diagnosis of NSTEMI was based on clinical symptoms, elevated troponin levels, echocardiographic findings, and ECG changes without persistent ST-segment elevation (or ST-segment elevation equivalents), according to the Fourth Universal Definition of Myocardial Infarction [1]. The inclusion criteria were: (1) fulfilment of diagnostic criteria for NSTEMI; (2) age ≥ 18 years; (3) performance of coronary angiography. The only exclusion criterion was the non-performance of coronary angiography; no additional exclusion criteria were applied.

**Definition of acute coronary occlusion:** ACO was defined as a culprit lesion with TIMI flow grade 0 during index coronary angiography. The culprit vessel was defined as the infarct-related artery, identified by the operator based on angiographic findings on index coronary angiography. Corresponding electrocardiographic findings and echocardiographic evidence of regional wall motion abnormalities (RWMA) were used as supportive findings but were not required for inclusion. Patients were divided into two groups: those meeting the aforementioned criteria, presenting with a culprit lesion with TIMI flow grade 0 on index coronary angiography constituted the ACO group, whereas those without complete occlusion (TIMI ≥ 1) were classified as non-ACO.

**Data collection:** Demographic, clinical, angiographic and procedural data were collected through the prospective Catheterisation Laboratory database of Attikon University Hospital, Athens, Greece. In terms of outcomes, in-hospital mortality data were also obtained. These data were subsequently used to compare baseline, clinical, and angiographic characteristics between the ACO and the non-ACO group. In addition, among patients with ACO, 12-lead ECGs, acquired at the time of admission, were retrospectively collected; ECGs from 7 patients were unavailable and could not be retrieved.

**AI-assisted ECG analysis:** Admission 12-lead ECGs from patients with angiographically confirmed ACO were retrospectively analysed using PMcardio (Queen of Hearts, Powerful Medical, Slovakia, v2.5), a clinically validated, CE-certified artificial intelligence-based ECG interpretation model [22]. This AI model accepts raw waveform data or converts ECG images to digitised waveforms. The model automatically provided key ECG parameters, including heart rate, rhythm, PR interval, QRS complex duration, corrected QT (QTc) interval, and ST/T-wave measurements. Then, the AI model classified each ECG into four diagnostic categories based on ECG patterns suggesting acute coronary occlusion. The diagnostic categories were STEMI, STEMI-equivalent, high-risk NSTEMI and no STEMI. In addition, the software provided an assessment of left ventricular systolic function derived from the ECG analysis.

Patients were further grouped according to the urgency of invasive management. Immediate invasive management corresponded to STEMI, STEMI equivalent and high-risk NSTEMI, whereas non-immediate invasive management corresponded to no STEMI.

**Statistical analysis:** Statistical analysis was performed to compare clinical and angiographic characteristics between groups. Continuous variables with normal distribution were expressed as mean ± standard deviation (SD) and were compared between groups using Welch’s *t*-test. Categorical variables were compared using the chi-square test or Fisher’s exact test. A *p*-value < 0.05 was considered statistically significant. Multivariable logistic regression analysis was performed to identify factors independently associated with angiographically confirmed ACO. Age, sex, hypertension, diabetes mellitus, current smoking, and dyslipidemia were included as covariates. The outcome variable was the presence of ACO, and results are reported as odds ratios (OR) with 95% confidence intervals (CI). All analyses were conducted using R software (v.4.5.2).

## 3. Results

**Figure 1:**
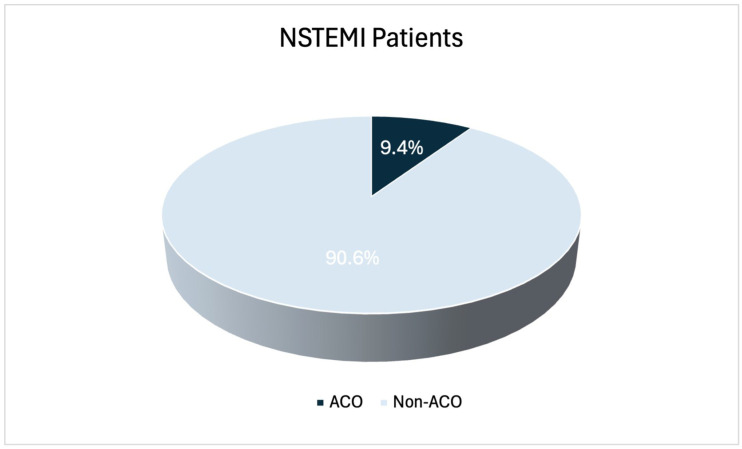
Prevalence of ACO among patients presenting with NSTEMI. ACO, acute coronary occlusion; NSTEMI, non–ST-segment elevation myocardial infarction.

Among 520 patients with NSTEMI, 49 (9.4%) were identified with angiographically confirmed acute coronary occlusion (Figure 1). Within the non-ACO group, 7.0% of patients had TIMI flow grade 1 on index coronary angiography (6.3% of the total population). Therefore, 15.7% of the study population had TIMI flow grade 0/1. Patients in the ACO group were younger compared to non-ACO patients (mean age 60.9 years ± 12.8 vs. 66.3 ± 12.0, *p* = 0.0065). There was no difference in sex distribution between the two groups (male sex 81.6% vs. 72.4%, *p* = 0.165). Regarding other baseline characteristics, no significant differences were observed in the presence of hypertension (53.1% vs. 63.9%, *p* = 0.135), diabetes mellitus (26.5% vs. 34.4%, *p* = 0.268), smoking (55.1% vs. 51.4%, *p* = 0.620) or family history (12.2% vs. 11.7%, *p* = 0.818) between the two groups. Dyslipidemia was the only clinical characteristic that differed significantly between groups, being more prevalent in the non-ACO group (38.8% vs. 53.9%, *p* = 0.043). Regarding the presence of previous PCI or CABG, no significant differences were observed between ACO and non-ACO patients (10.2% vs. 19.3%, *p* = 0.173; 0.0% vs. 7.4%, *p* = 0.064, respectively). Echocardiographic assessment showed no significant difference in left ventricular systolic dysfunction (LVEF < 40%) between the two groups (18.4% vs. 15.9%, *p* = 0.658).

**Figure 2:**
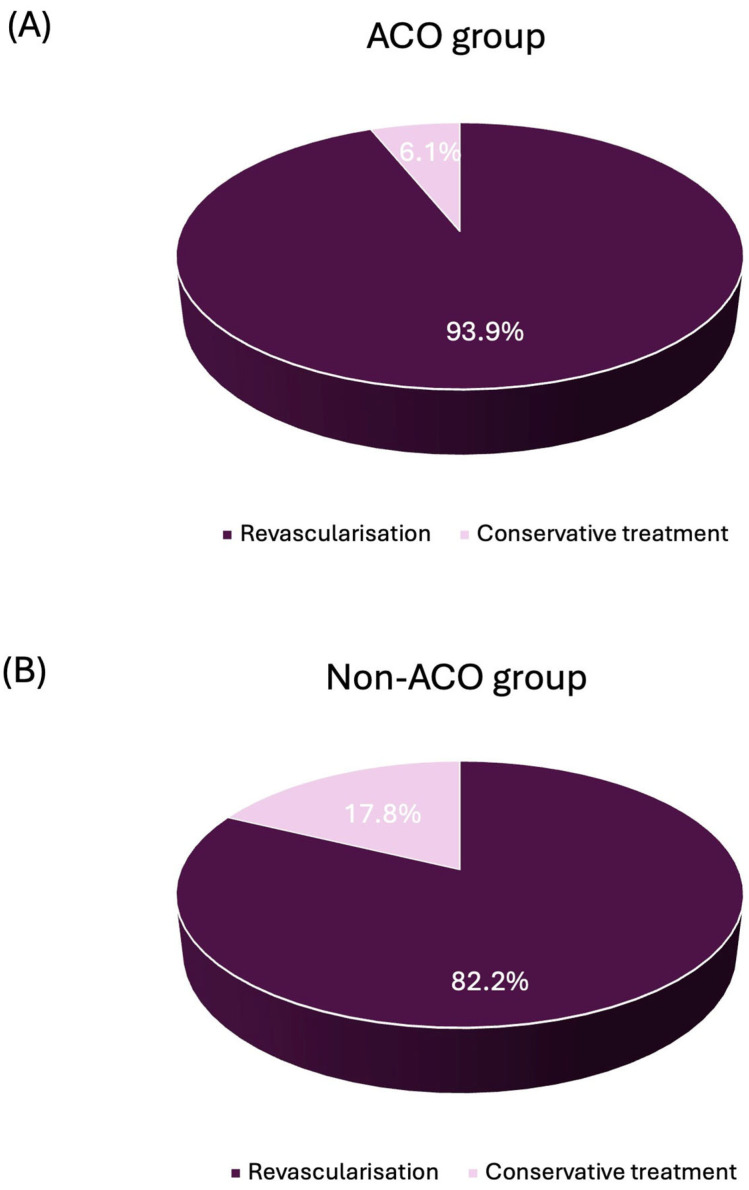
Management strategy (revascularisation vs. conservative treatment) in NSTEMI patients with and without acute coronary occlusion. (**A**) Patients with ACO. (**B**) Patients without ACO. ACO, acute coronary occlusion; NSTEMI, non–ST-segment elevation myocardial infarction.

Notably, revascularisation was performed significantly more often in ACO patients compared to non-ACO patients (93.9% vs. 82.2%, *p* = 0.037) (Figure 2). In both groups, revascularisation was mainly achieved through percutaneous coronary intervention (PCI). Furthermore, angiographic findings revealed marked differences in the distribution of culprit vessels between the two groups. In the ACO group, the left circumflex artery (LCX) was the predominant culprit vessel (49.0%), followed by the right coronary artery (RCA) (34.7%) and the left anterior descending artery (LAD) (16.3%), whereas in the non-ACO group, LAD was the predominant culprit vessel (52.3%), followed by the LCX (27.0%) and the RCA (18.4%) (*p* < 0.0001). In terms of short-term outcomes, in-hospital mortality was low and did not differ between the two groups (ACO 2.0% vs. non-ACO 3.0%, *p* > 0.99). Furthermore, during hospitalisation, none of the patients experienced reinfarction or required repeat revascularisation. Baseline, clinical and angiographic characteristics are presented in Table 1.

In multivariable logistic regression analysis, age was independently associated with angiographically confirmed acute coronary occlusion (OR 0.96, 95% CI 0.93–0.99, *p* = 0.007). No other cardiovascular risk factor, including sex (OR 1.63, 95% CI 0.79–3.72, *p* = 0.214), hypertension (OR 0.94, 95% CI 0.50–1.80, *p* = 0.854), diabetes mellitus (OR 0.94, 95% CI 0.45–1.90, *p* = 0.872), smoking status (OR 0.69, 95% CI 0.35–1.36, *p* = 0.280) or dyslipidemia (OR 0.56, 95% CI 0.29–1.04, *p* = 0.071), was independently associated with the presence of ACO. Results are summarised in Table 2.

**Figure 3:**
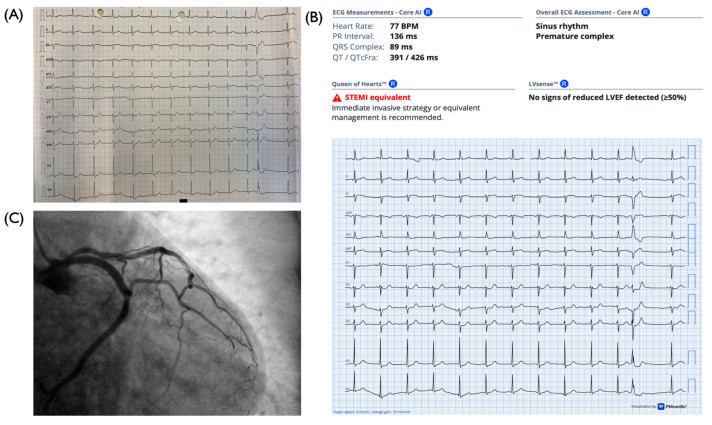
Representative example of a NSTEMI patient with ACO. (**A**) Admission 12-lead electrocardiogram of the patient. (**B**) AI-assisted ECG interpretation showing a STEMI-equivalent pattern; no signs of reduced LVEF were detected. (**C**) Corresponding coronary angiography confirms acute coronary occlusion. ACO, acute coronary occlusion; AI, artificial intelligence; ECG, electrocardiogram; LVEF, left ventricular ejection fraction.

AΙ-assisted ECG interpretation was performed on 42 out of the 49 admission 12-lead ECGs from patients with angiographically confirmed ACO (Figure 3). The results showed that 2.4% of ECGs were classified as STEMI, 31.0% as STEMI-equivalent, 23.8% as high-risk NSTEMI, and 42.9% as no STEMI. Overall, 57.1% of cases were identified as requiring immediate invasive management, corresponding to the combined categories of STEMI, STEMI equivalent and high-risk NSTEMI. Among the remaining 42.9%, not classified as requiring immediate invasive management, 38.9% showed reduced left ventricular ejection fraction (LVEF < 40%) based on ECG analysis. Taken together, 73.8% of patients exhibited either an indication for immediate invasive management or evidence of significantly impaired left ventricular systolic function, findings that would support an expedited referral to the catheterisation laboratory.

## 4. Discussion

The present study demonstrates that a significant proportion of patients classified as NSTEMI present with acute coronary occlusion despite the absence of diagnostic ST-segment elevation on their initial ECG. Specifically, 9.4% of NSTEMI patients in our cohort exhibited ACO, suggesting that the traditional STEMI/NSTEMI classification may not always reliably identify the occlusion. Within the non-ACO group, 7.0% of patients had TIMI flow grade 1 on index coronary angiography (corresponding to 6.3% of the total population), resulting in a combined proportion of 15.7% of patients with TIMI flow grade 0/1. These findings align with prior studies reporting a considerable proportion of NSTEMI patients with ACO [5,7,9,28]. Khan et al. conducted a meta-analysis including 40,777 patients and indicated that 25.5% of NSTEMI patients had ACO on coronary angiography [5]. Similarly, Yu et al. reported a proportion of 25.4% of NSTEMI patients with ACO [29], while other studies reported a prevalence of 29.3% and 14.9%, respectively [30,31]. All the aforementioned studies used broader angiographic inclusion criteria compared to ours, including lesions with TIMI flow grade 0–1, 0–2, and in some cases, TIMI flow grade 3; in contrast, ACO in our study was strictly defined as a culprit lesion with TIMI flow grade 0, which likely explains the lower proportion of ACO in our population. However, in our study, the combined proportion of patients with TIMI flow grade 0/1 accounted for 15.7%, consistent with prior studies using this definition. Furthermore, in our institution, relatively liberal criteria are applied for primary PCI pathway activation to reduce the risk of missing true STEMIs, which may also explain the lower proportion of ACO observed in our cohort compared with previous studies. Additionally, in line with our findings, previous investigations also reported a predominance of the LCX as the culprit vessel in NSTEMI patients with ACO [22,29,32].

In our cohort, in-hospital mortality was low and did not differ significantly between NSTEMI patients with and without ACO (2.0% vs. 3.0%, respectively, *p* > 0.99). In contrast, previous studies have reported worse clinical outcomes among NSTEMI patients presenting with ACO [5,6,7,9,29,33,34,35,36]. The relatively small size of our cohort might explain the difference in outcomes compared to previous studies. More precisely, in the meta-analysis by Khan et al., patients with NSTEMI and ACO had significantly higher rates of MACE and increased all-cause mortality [5], while other investigators further indicated a higher frequency of cardiogenic shock and increased mortality among NSTEMI ACO patients compared to non-ACO patients [30]. Furthermore, Herman et al., in a large retrospective study of 9943 patients, reported higher all-cause mortality in NSTEMI patients with ACO compared to those without ACO. They also observed a significantly longer delay to coronary angiography and reperfusion in this population [8]. Increased door-to-balloon time in NSTEMI patients with ACO is also reported in recent studies [7,19,22], underscoring the need for more refined diagnostic algorithms in acute coronary syndrome management.

Artificial intelligence-assisted ECG interpretation has emerged as a promising tool for earlier identification of ACO in patients with myocardial infarction [12,21,22,23,26,27,37,38]. In our study, the AI model successfully identified more than half of ACO cases, whereas among those not classified as requiring immediate intervention, impaired left ventricular systolic function was frequently detected. These findings suggest that AI-assisted ECG analysis may recognise subtle ischaemic abnormalities that escape visual interpretation. Previous studies have demonstrated that AI-based ECG algorithms may achieve higher sensitivity for the detection of angiographically confirmed ACO compared with conventional STEMI criteria [12,21,22]. In addition, recent evidence suggests that the use of an AI-assisted ECG interpretation model may improve timely reperfusion by reducing diagnostic and treatment delays [37]. In our study, 38.9% of patients not classified as requiring immediate intervention showed reduced left ventricular ejection fraction on AI analysis. This observation indicates that AI-detected reduced LVEF may reveal subtle functional impairment, potentially indicating the need for further evaluation in the catheterisation laboratory. While additional studies are required to define the role of AI-LVEF assessment in clinical practice, it may provide a sign to clinicians, encouraging reassessment or closer monitoring. However, these findings should be interpreted as hypothesis-generating and exploratory, given the limited sample size and the assessment of AI analysis being restricted to ACO cases only. Therefore, the clinical utility of AI-assisted ECG interpretation remains to be established, and further studies are required to validate these observations and define their diagnostic performance.

### 4.1. Clinical Implications and Future Perspectives

The present study has clinical implications for the future management of acute coronary syndromes. Recognition that a substantial proportion of patients classified as NSTEMI present with ACO supports a shift toward an occlusion-oriented approach, irrespective of ST-segment elevation patterns, which can potentially improve early identification of high-risk patients who could benefit from expedited invasive management. Accurate ECG-based detection of ACO at first medical contact, through AI-assisted ECG interpretation models, may facilitate more appropriate triage and improved risk stratification, thereby leading to timely reperfusion with a potentially favourable impact on clinical outcomes.

Future prospective multicenter studies are warranted to validate these findings, determine their impact on clinical outcomes and clarify how AI models can be optimally integrated into real-world clinical practice. Ultimately, combining clinical and imaging assessment with AI-assisted ECG interpretation may contribute to a more precise, individualised management strategy for AMI.

### 4.2. Limitations

This study has several limitations. Firstly, it was conducted in a single tertiary centre, which may limit the generalizability of the findings to other populations or healthcare settings. Secondly, contrary to other studies, only patients with TIMI flow 0 were included in the ACO group. However, it could be argued that patients with TIMI flow 1 or even 2 should be differentiated and treated differently from patients with normal coronary flow. Therefore, our definition of ACO may slightly underestimate the number of NSTEMI patients with acute occlusion, particularly those with residual antegrade flow (TIMI 1–2). Furthermore, in our institution, relatively liberal criteria are applied for primary PCI pathway activation to reduce the risk of missing true STEMIs, which may also explain the lower proportion of ACO observed in our cohort compared with previous studies. Additionally, potential inter-observer variability in angiographic interpretation cannot be completely excluded. The artificial intelligence–based ECG analysis was performed retrospectively and in a relatively small subset of patients, which may introduce selection or ascertainment bias and limit statistical power, respectively. Furthermore, the study did not include the performance of the AI model in the non-ACO group, which may limit the assessment of its specificity. Finally, the study did not include long-term clinical outcomes or short-term clinical outcome measures, such as time to angiography, time to revascularisation, cardiogenic shock, ventricular arrhythmias, or worsening heart failure.

## 5. Conclusions

A significant proportion of NSTEMI patients present with ACO, indicating the limitations of the traditional STEMI/NSTEMI classification and underscoring the need for emerging diagnostic strategies. Artificial intelligence-assisted ECG interpretation may represent an exploratory tool for earlier recognition of ACO, pending further validation. Future studies are warranted to explore the optimal management of AMI beyond the traditional ST-segment elevation classification.

## Figures and Tables

**Table 1:** Baseline, clinical and angiographic characteristics of NSTEMI patients with and without ACO.

Characteristics	ACO (n = 49)	Non-ACO (n = 471)	*p*-Value
Age, years (mean ± SD)	60.9 ± 12.8	66.3 ± 12.0	0.0065
Sex, male	40 (81.6%)	341 (72.4%)	0.165
Hypertension	26 (53.1%)	301 (63.9%)	0.135
Diabetes mellitus	13 (26.5%)	162 (34.4%)	0.268
Current smoker	27 (55.1%)	242 (51.4%)	0.620
Dyslipidemia	19 (38.8%)	254 (53.9%)	0.043
Family history	6 (12.2%)	55 (11.7%)	0.818
Impaired Renal function (Cr > 2.1 mg/dL)	0 (0.0%)	26 (5.5%)	0.158
Atrial fibrillation	5 (10.2%)	44 (9.3%)	0.798
Paroxysmal	3 (6.1%)	24 (5.1%)	
Chronic	2 (4.1%)	20 (4.2%)	
Previous PCI	5 (10.2%)	91 (19.3%)	0.173
Previous CABG	0 (0.0%)	35 (7.4%)	0.064
LVEF < 40% (Echocardiography)	9 (18.4%)	75 (15.9%)	0.658
Revascularisation	46 (93.9%)	387 (82.2%)	0.037
PCI	45 (91.8%)	313 (66.5%)	
CABG	1 (2.0%)	74 (15.7%)	
Culprit vessel			<0.0001
LMCA	0/49 (0.0%)	7/304 (2.3%) *	
LAD	8/49 (16.3%)	159/304 (52.3%) *	
LCX	24/49 (49.0%)	82/304 (27.0%) *	
RCA	17/49 (34.7%)	56/304 (18.4%) *	

* Culprit vessel distribution was reported only in patients with a clearly identified culprit lesion, as determined by the operator. Abbreviations: ACO, acute coronary occlusion; LVEF, left ventricular ejection fraction; Cr, creatinine; PCI, percutaneous coronary intervention; CABG, coronary artery bypass grafting; LMCA, left main coronary artery; LAD, left anterior descending artery; LCX, left circumflex artery; RCA, right coronary artery.

**Table 2:** Multivariable logistic regression analysis for ACO.

Variable	OR	95% CI	*p*-Value
Age	0.96	0.93–0.99	0.007
Male sex	1.63	0.79–3.72	0.214
Hypertension	0.94	0.50–1.80	0.854
Diabetes mellitus	0.94	0.45–1.90	0.872
Current smoking	0.69	0.35–1.36	0.280
Dyslipidemia	0.56	0.29–1.04	0.071

Abbreviations: ACO, acute coronary occlusion; OR, Odds Ratio; CI, Confidence Interval.

## Data Availability

The data presented in this study are available on request from the corresponding author.

## Abbreviations

The following abbreviations are used in this manuscript:

ECG	Electrocardiogram
ACO	Acute coronary occlusion
AI	Artificial intelligence
AMI	Acute myocardial infarction
STEMI	ST-elevation myocardial infarction
NSTEMI	Non-ST-elevation myocardial infarction
MACE	Major adverse cardiac events
ACC	American College of Cardiology
TIMI	Thrombolysis in Myocardial Infarction
SD	Standard deviation
OR	Odds ratio
CI	Confidence interval
PCI	Percutaneous coronary intervention
LCX	Left circumflex artery
RCA	Right coronary artery
LAD	Left anterior descending artery
LVEF	Left ventricular ejection fraction
Cr	Creatinine
LMCA	Left main coronary artery
CABG	Coronary artery bypass grafting
